# Heat Transfer Attributes of Gold–Silver–Blood Hybrid Nanomaterial Flow in an EMHD Peristaltic Channel with Activation Energy

**DOI:** 10.3390/nano12101615

**Published:** 2022-05-10

**Authors:** Basma Souayeh, Katta Ramesh, Najib Hdhiri, Essam Yasin, Mir Waqas Alam, Kawthar Alfares, Amina Yasin

**Affiliations:** 1 Department of Physics, College of Science, King Faisal University, P.O. Box 400, Al-Ahsa 31982, Saudi Arabia; wmir@kfu.edu.sa (M.W.A.); kalfares@kfu.edu.sa (K.A.); 2 Laboratory of Fluid Mechanics, Physics Department, Faculty of Sciences of Tunis, University of Tunis El Manar, Tunis 2092, Tunisia; hdhiri_najib@yahoo.fr; 3 Department of Mathematics, Symbiosis Institute of Technology, Symbiosis International (Deemed University), Pune 412115, India; ramesh.katta1@gmail.com; 4 Department of Mathematics, Statistics and Physics, College of Arts and Science, University of Qatar, Doha P.O. Box 2713, Qatar; essamyasin@qu.edu.qa; 5 Department of Basic Sciences, Preparatory Year Deanship, King Faisal University, Al Hofuf 31982, Saudi Arabia; ayasin@kfu.edu.sa

**Keywords:** hybrid nanofluid, bioconvection, activation energy, gyrotactic microorganisms, gold and silver nanoparticles, physiological flow

## Abstract

The heat enhancement in hybrid nanofluid flow through the peristaltic mechanism has received great attention due to its occurrence in many engineering and biomedical systems, such as flow through canals, the cavity flow model and biomedicine. Therefore, the aim of the current study was to discuss the hybrid nanofluid flow in a symmetric peristaltic channel with diverse effects, such as electromagnetohydrodynamics (EMHD), activation energy, gyrotactic microorganisms and solar radiation. The equations governing this motion were simplified under the approximations of a low Reynolds number (LRN), a long wavelength (LWL) and Debye–Hückel linearization (DHL). The numerical solutions for the non-dimensional system of equations were tackled using the computational software Mathematica. The influences of diverse physical parameters on the flow and thermal characteristics were computed through pictorial interpretations. It was concluded from the results that the thermophoresis parameter and Grashof number increased the hybrid nanofluid velocity near the right wall. The nanoparticle temperature decreased with the radiation parameter and Schmidt number. The activation energy and radiation enhanced the nanoparticle volume fraction, and motile microorganisms decreased with an increase in the Peclet number and Schmidt number. The applications of the current investigation include chyme flow in the gastrointestinal tract, the control of blood flow during surgery by altering the magnetic field and novel drug delivery systems in pharmacological engineering.

## 1. Introduction

In the current century, it has been identified that the flow of nanofluids in various geometries is a potential research area. This is due to the researchers who have ameliorated the performance of heat transfer and other characteristics by adding different nanoparticles into pure base fluids, which leads to considerable practical applications in diverse situations, such as biomedical engineering, cancer treatment thermal therapy, microelectronics, metallurgical sectors, the delivery of drugs, microchannels, power generation and micromanufacturing processes. Initially, Choi [1] introduced the concept of nanofluids for coolants and cooling purposes in industry. The nanoparticles are typically metals, carbides and oxides and the base fluids comprise ethanol, ethylene glycol, oil, blood and water. Out of these nanoparticles, silver and gold have received the most consideration in research due to their realistic applications in cancer treatments, sterilization techniques and several other areas. Mahian et al. [2] presented a comprehensive review of the applications of nano-liquid flow. Okonkwo et al. [3] delivered a detailed updated review of nano-liquids in diverse heat transfer devices. Aman et al. [4] provided perturbation results for the propulsion of gold–silver nano-liquid through parallel plates. Baber et al. [5] discussed the synthesis of silver and gold nanoparticles in a coaxial propulsion reactor. Hussain et al. [6] studied the multiphase motion of Casson gold liquid in a steep channel. Mondragón [7] reported on the synthesis and characterization of gold/water nanofluids, which have applications in industry. Eid et al. [8] presented the Runge–Kutta–Fehlberg scheme solutions to discuss the energy features of nano-liquid flow with a suspension of gold nanoparticles in a base fluid over a stretching surface. Suleman et al. [9] used a shooting technique to analyze the results of Ag/water nanofluid propulsion over a cylinder. Waghole et al. [10] provided an experimental investigation on the propulsion of silver nano-liquid in a tube with twisted tape inserts. Pourhoseini et al. [11] performed theoretical and experimental investigations on the heat transfer performance of a plate heat exchanger with the effect of silver/water nanofluid. Forghani-Tehrani et al. [12] presented finite volume solutions for the motion of water–silver nanofluid in a microchannel. Ali et al. [13] discussed the 3D flow of nanofluid over a stretching sheet using finite element analysis. Benos et al. [14] investigated carbon nanotube flow in a 2D shallow cavity. Gkountas et al. [15] studied the impact of nanofluid on the Printed Circuit Heat Exchanger (PCHE’s) thermal-hydraulic performance. Benos and Sarris [16] studied the 2D magnetohydrodynamic (MHD) natural convection of a nanofluid-filled shallow cavity.

Choi perhaps was the first to work on nanofluids for industrial applications; however, his research and many of the previously mentioned works were confined to unitary nanofluids (nanofluids with a single nanoparticle type). Later in 2004, Makishima et al. [17] propounded suspensions of nanofluids encompassing different nanoparticles merged in a base fluid to dispense the homogenous phase, which they named hybrid nano-liquids. It has been noticed in many investigations that hybrid nanofluids have received enhanced thermal conductivity characteristics compared with typical nano-liquids. The addition of hybrid nanoparticles to the base fluid results a significant enhancement of thermal conductivity. Researchers are not in the position to exercise the hybrid nano-liquids since there are a great deal of problems that must be resolved for the use of hybrid nano-liquids in domestic and industrial applications. Many investigators have taken on the responsibility of studying the flow of hybrid nanofluids in diverse geometries in the direction of real-world applications; however, they are very few. A smaller number of articles have been devoted to hybrid nano-liquids. Zhang et al. [18] used a bvp4c scheme to study the motion of a 3D hybrid nano-liquid over a disk and concluded that the performance of the hybrid nano-liquid was better than the conventional nanofluid. Shoaib et al. [19] used the Lobatto IIIA method to establish the results of the motion of a radiative 3D water-based hybrid nano-liquid over a sheet. Ahmad et al. [20] carried out a numerical investigation on the propulsion of graphene oxide, silver–kerosene oil and graphene oxide–kerosene oil hybrid nano-liquids over a sheet. Gamachu and Ibrahim [21] discussed the motion of a viscoelastic hybrid nano-liquid over a disk by considering aluminum oxide and silver in carboxymethyl cellulose water. Yarmand et al. [22] presented their experimental work on the motion of silver–graphene nanoplatelets–water hybrid nanofluids in a tube. Hayat and Nadeem [23] reported numerical results for the rotating motion of a hybrid nano-liquid with silver and copper oxide nano-sized particles. Dinarvand and Nadem [24] studied the motion of an aqueous gold–zinc oxide hybrid nano-liquid over a disk using the finite difference method. Dinarvand et al. [25] discussed the flow of a silver–magnesium oxide–water hybrid nano-liquid in a slim needle using the finite difference method. Kot and Elmaboud [26] carried out a numerical analysis on the propulsion of hybrid nano-liquid flows in a diseased artery. A few more studies can be seen in [27,28,29,30,31].

Nowadays, research on bioconvection has received a great deal of attention due to its tremendous applications in electronic, civil, process, mechanical and chemical engineering. More specifically, bioconvection applications include cooling systems, building insulation, microreactors, micro-heat pipes and micro-channel thermal sinks. In physiological systems, bioconvection is involved in biomedical instrumentation, nano-biotechnology, microenzymes, biosensors, blood flow, nanomedicine, content detection, pharmacokinetics and drug delivery. In the past few decades, the implementation of activation energy has been much considered in some engineering advances. For instance, thermal magnetic flux, fusion control, nuclear reactor cooling, liquid metal filtration, casting and compact heat exchangers. In view of the applications of bioconvection and activation energy, several authors have started working on these crucial topics. Rekha [32] et al. discussed the motion of nanofluid in a plate, wedge and cone with a suspension of aluminum alloys as nanoparticles in water as a base fluid and activation energy. Yusuf et al. [33] analyzed the effects of bioconvection and activation energy on the motion of Williamson nano-liquid past a stretchy plate. Punith Gowda et al. [34] investigated the impacts of activation energy on the boundary layer flow of a nanofluid with the Runge–Kutta–Fehlberg scheme. Khan et al. [35] studied Darcy–Forchheimer hybrid nanofluid flow over a stretchable surface with an activation energy.

The phenomenon of peristalsis is a well-known mechanism in physiological systems. It is a vital and automatic process that drives the biological liquids in the fallopian tube, duodenum, reproductive tract and many other situations. The concept of this phenomenon is exploited in many bioengineering and industrial processes, such as novel pharmacological delivery systems, sanitary fluid transport, corrosive fluid transport and blood pumps in heart–lung machines. The propulsion of hybrid nanofluids in biological systems has great importance in medical sciences and engineering. Its applications include cancer therapy and blood circulation. There is also some less well-known work on hybrid nanofluid flow through peristaltic geometry. For instance, Bhatti and Abdelsalam [36] discussed Carreau fluid flow through peristalsis using hybrid nanoparticles of tantalum and gold. Bibi and Xu [37] studied magnetohydrodynamic Carreau hybrid nano-liquid flow with silver and copper nanoparticles in a bio-channel using homotopy-based package-BVPh 2.0. McCash et al. [38] carried out a theoretical investigation on the peristaltic propulsion of a hybrid nano-liquid (Cu–Ag–water) in a peristaltic duct. Das et al. [39] used Mathematica to discuss the motion of an ionic Casson hybrid nano-liquid (silver–silicon dioxide/pure water) in a micro-peristaltic channel. Awais et al. [40] provided homotopy results for the propulsion of power-law hybrid nanofluids in a ciliated peristaltic tube by considering titanium dioxide and silver nanoparticles.

In the earlier literature, authors have presented their analysis on nanofluid and hybrid nanofluid flows in various geometries, and a very few works exist in the direction of hybrid nanofluid flows in a peristaltic mechanism. We also noticed that no work has been made in the direction of hybrid nano-liquid (gold–silver–water) propulsion in a bio-channel. With this motivation, the current article deals with the flow of a hybrid nanofluid through a symmetric channel with the effects of solar radiation, electromagnetohydrodynamics, gyrotactic microorganisms and activation energy. The non-dimensional problem is solved with the computational software Mathematica. The numerical solutions are presented in pictorial and tabular forms for velocity, nanoparticle temperature, nanoparticle concentration, motile microorganisms, Sherwood number, Nusselt number and skin friction. The current results have many applications in bio-medical and engineering fields, including in DNA analyzers, water filtration and purification processes, microchannel devices and electro-osmotic pumps.

## 2. Mathematical Model

**Figure 1:**
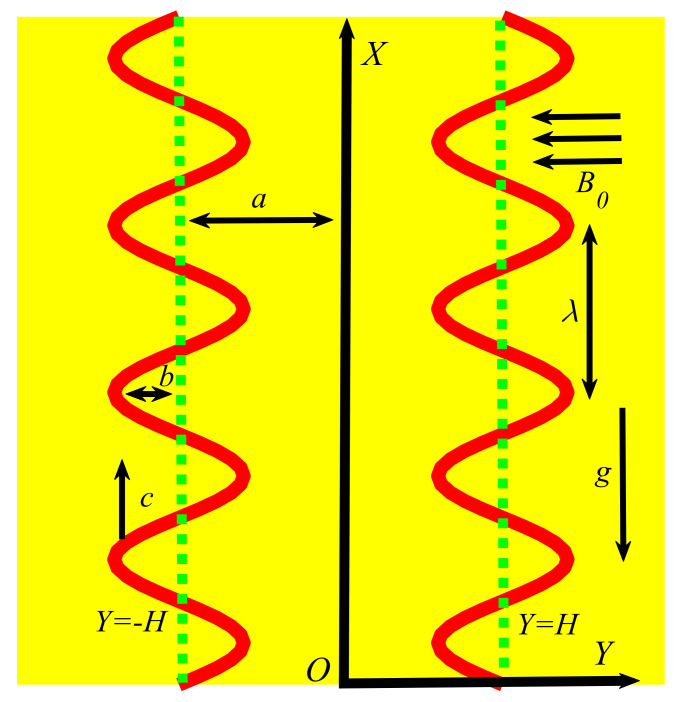
Flow situation of the hybrid nanofluids through peristalsis.

Consider the motion of a hybrid nanofluid (gold–silver–water) through an electromagnetohydrodynamic peristaltic channel under the effects of activation energy, solar radiation and gyrotactic microorganisms. The Cartesian coordinate system (X,Y) is adopted in such a way that the X axis is in the flow direction and the Y axis is in the transverse direction of the fluid flow. The flow of the hybrid nanofluid is assumed to be due to the peristalsis and electro-osmosis. A uniform magnetic field $B_{0}$ is applied in the transverse direction of actual fluid motion. It is assumed that the right wall of the channel is maintained at a temperature $T_{0}$ and a concentration $C_{0}$. The temperature and concentrations of the left wall can be assumed as $T_{1}$ and $C_{1}$ (see Figure 1). The representation of the channel walls is written as follows [41]:

(1) $$Y=\pm H(X,t)=\pm \left(a+b\;\sin\left(\frac{\pi }{\lambda }\left(X-ct\right)\right)\right),$$

in which H is the channel wall, a defines the channel half width, b denotes the wave amplitude, c is the wave speed, t is the time and λ denotes the wavelength.

The equations (continuity, momentum, energy, nanoparticle concentration and microorganisms) governing the flow of the hybrid nanofluid in the laboratory frame can be written as follows [42,43,44]:

(2) $$\frac{\partial U}{\partial X}+\frac{\partial V}{\partial Y}=0,$$

(3) $$\begin{array}{l} \rho_{hnf}\left(\frac{\partial U}{\partial t}+U\frac{\partial U}{\partial X}+V\frac{\partial U}{\partial Y}\right)=-\frac{\partial P}{\partial X}+\mu_{hnf}\left(\frac{\partial^{2}U}{\partial X^{2}}+\frac{\partial^{2}U}{\partial Y^{2}}\right)-\sigma_{hnf}B_{0}{}^{2}U+\rho_{e}E_{x}\\ \;\;\;\;\;\;\;\;\;\;\;\;\;\;\;\;\;\;\;\;\;\;\;\;+{\left(\rho \beta \right)}_{hnf}g\left(1-C_{0}\right)\left(T-T_{0}\right)-g\left(\rho_{p}-\rho_{f}\right)\left(C-C_{0}\right)-g\gamma \left(\rho_{m}-\rho_{f}\right)\left(N-N_{0}\right)\;, \end{array}$$

(4) $$\rho_{hnf}\left(\frac{\partial V}{\partial t}+U\frac{\partial V}{\partial X}+V\frac{\partial V}{\partial Y}\right)=-\frac{\partial P}{\partial Y}+\mu_{hnf}\left(\frac{\partial^{2}V}{\partial X^{2}}+\frac{\partial^{2}V}{\partial Y^{2}}\right)\;,$$

(5) $$\begin{array}{l} {\left(\rho c_{p}\right)}_{hnf}\left(\frac{\partial T}{\partial t}+U\frac{\partial T}{\partial X}+V\frac{\partial T}{\partial Y}\right)=\left(k_{hnf}+\frac{16\sigma^{*}T_{0}{}^{3}}{3k^{*}}\right)\left(\frac{\partial^{2}T}{\partial X^{2}}+\frac{\partial^{2}T}{\partial Y^{2}}\right)\\ \;\;\;\;\;\;\;\;\;\;\;\;\;\;\;\;\;\;\;\;\;\;\;\;\;\;\;\;\;\;\;\;\;\;\;\;\;\;\;\;\;\;\;\;\;\;\;\;\;\;+{\left(\rho c_{p}\right)}_{p}\left(D_{B}\left(\frac{\partial C}{\partial X}\frac{\partial T}{\partial X}+\frac{\partial C}{\partial Y}\frac{\partial T}{\partial Y}\right)+\frac{D_{T}}{T_{m}}\left({\left(\frac{\partial T}{\partial X}\right)}^{2}+{\left(\frac{\partial T}{\partial Y}\right)}^{2}\right)\right)\;, \end{array}$$

(6) $$\frac{\partial C}{\partial t}+U\frac{\partial C}{\partial X}+V\frac{\partial C}{\partial Y}=D_{B}\left(\frac{\partial^{2}C}{\partial X^{2}}+\frac{\partial^{2}C}{\partial Y^{2}}\right)+\frac{D_{T}}{T_{m}}\left(\frac{\partial^{2}T}{\partial X^{2}}+\frac{\partial^{2}T}{\partial Y^{2}}\right)-k_{r}{}^{2}\left(C-C_{0}\right){\left(\frac{T}{T_{0}}\right)}^{n}\exp\;\;\left(\frac{-E_{a}}{\omega T}\right)\;,$$

(7) $$\frac{\partial N}{\partial t}+U\frac{\partial N}{\partial X}+V\frac{\partial N}{\partial Y}+\frac{b^{*}W_{e}}{\left(C_{1}-C_{0}\right)}\left(\frac{\partial }{\partial X}\left(N\frac{\partial C}{\partial X}\right)+\frac{\partial }{\partial Y}\left(N\frac{\partial C}{\partial Y}\right)\right)=D_{m}\left(\frac{\partial^{2}N}{\partial X^{2}}+\frac{\partial^{2}N}{\partial Y^{2}}\right)\;.$$

with the following corresponding boundary conditions [45,46]:

(8) $$U=0,T=T_{0},C=C_{0},N=N_{0}\;\mathrm{at}\;Y=H,$$

(9) $$U=0,T=T_{1},C=C_{1},N=N_{1}\;\mathrm{at}\;Y=-H,$$

where, *U* and *V* are the velocity components, $\rho_{hnf}$ is the effective density of the hybrid nanofluid, $\mu_{hnf}$ is the dynamic viscosity of the hybrid nanofluid, $\sigma_{hnf}$ is the electrical conductivity of the hybrid nanofluid, ${\left(\rho \beta \right)}_{hnf}$ is the effective thermal expansion, g is the gravitational force, T is the nanoparticle temperature, $\rho_{p}$ is the nanoparticle density, $\rho_{f}$ is the base fluid density, n is the fitted rate, $n_{0}$ is the concentration of motile organisms, γ is the ambient volume of microorganisms, $\rho_{m}$ is the density of motile organisms, $\rho_{e}$ is the electrical charge density, $E_{x}$ is the electric field, ${\left(\rho c_{p}\right)}_{hnf}$ is the effective heat capacity of the hybrid nanofluid, $k_{hnf}$ is the thermal diffusivity of the hybrid nanofluid, $\sigma^{*}$ is the Stefan–Boltzmann constant, $k^{*}$ is the mean absorption coefficient, $D_{B}$ is the Brownian diffusion coefficient, C is the nanoparticle volume fraction, $D_{T}$ is the thermophoretic diffusion coefficient, $T_{m}$ is the mean temperature, $k_{r}$ is the rate of the reaction, N is the motile microorganism, $E_{a}$ is the activation energy, ω is the Boltzmann constant, $b^{*}$ is the chemotaxis constant, $W_{e}$ is the swimming cell speed, P is the pressure and $D_{m}$ is the diffusion coefficient of the microorganisms.

The thermophysical properties of water and hybrid nanofluids with various shapes are given by the following equations [47]:

(10) $$\frac{\mu_{hnf}}{\mu_{f}}=\frac{1}{{\left(\;\left(1-\varphi_{1}\right)\left(1-\varphi_{2}\right)\right)}^{2.5}}\;,$$

(11) $$\frac{\rho_{hnf}}{\rho_{f}}=\left(\left(1-\varphi_{2}\right)\left(1-\varphi_{1}+\frac{\varphi_{1}\rho_{1}}{\rho_{f}}\right)\right)+\frac{\varphi_{2}\rho_{2}}{\rho_{f}}\;,$$

(12) $$\frac{{\left(\rho c_{p}\right)}_{hnf}}{{\left(\rho c_{p}\right)}_{f}}=\left(\left(1-\varphi_{2}\right)\left(1-\varphi_{1}+\frac{\varphi_{1}{\left(\rho c_{p}\right)}_{1}}{{\left(\rho c_{p}\right)}_{f}}\right)\right)+\frac{\varphi_{2}{\left(\rho c_{p}\right)}_{2}}{{\left(\rho c_{p}\right)}_{f}}\;,$$

(13) $$\frac{{\left(\rho \beta \right)}_{hnf}}{{\left(\rho \beta \right)}_{f}}=\left(\left(1-\varphi_{2}\right)\left(1-\varphi_{1}+\frac{\varphi_{1}{\left(\rho \beta \right)}_{1}}{{\left(\rho \beta \right)}_{f}}\right)\right)+\frac{\varphi_{2}{\left(\rho \beta \right)}_{2}}{{\left(\rho \beta \right)}_{f}}\;,$$

(14) $$\sigma_{hnf}=\left(\frac{\sigma_{2}\left(1+2\varphi_{2}\right)+2\sigma_{bf}\left(1-\varphi_{2}\right)}{\sigma_{2}\left(1-\varphi_{2}\right)+\sigma_{bf}\left(2+\varphi_{2}\right)}\right)\sigma_{bf}\;;\;\;\;\sigma_{bf}=\left(\frac{\sigma_{1}\left(1+2\varphi_{1}\right)+2\sigma_{f}\left(1-\varphi_{1}\right)}{\sigma_{1}\left(1-\varphi_{1}\right)+\sigma_{f}\left(2+\varphi_{1}\right)}\right)\sigma_{f}\;,$$

(15) $$k_{hnf}=\left(\frac{k_{2}+\left(m-1\right)k_{bf}-\left(m-1\right)\varphi_{2}\left(k_{bf}-k_{2}\right)}{k_{2}+\left(m-1\right)k_{bf}+\varphi_{2}\left(k_{bf}-k_{2}\right)}\right)k_{bf}\;;\;\;\;k_{bf}=\left(\frac{k_{1}+\left(m-1\right)k_{f}-\left(m-1\right)\varphi_{1}\left(k_{f}-k_{1}\right)}{k_{1}+\left(m-1\right)k_{f}+\varphi_{1}\left(k_{f}-k_{1}\right)}\right)k_{f\;.}$$

Consider the following transformations between the wave and fixed frame:

(16) $$x=X-ct\;,\;\;\;\;\;y=Y\;,\;\;\;\;\;u=U-c\;,\;\;\;\;\;v=V\;,\;\;\;\;\;p=P\;,\;\;\;\;\;\bar{N}=N\;,\;\;\;\;\;\bar{T}=T\;,\;\;\;\;\;\bar{C}=C.$$

We can introduce the non-dimensional variables as follows:

u¯=uc ,     v¯=vcδ ,    x¯=xλ ,    y¯=ya ,    p¯=a2pcλμf ,    θ=T¯−T0T1−T0 ,    σ=C¯−C0C1−C0 ,    χ=N¯−N0N1−N0 ,    M=σfμfB0a ,    Re=ρfcaμf ,    δ=aλ ,     ε=ba ,     Rb=(ρm−ρf) γ (N1−N0)(ρβ)f (1−C0) (T1−T0) ,    Gr=g(ρβ)f (1−C0) (T1−T0)a2cμf ,    Nr=(ρm−ρf) (C1−C0)(ρβ)f (1−C0) (T1−T0) ,    Rn=16σ*T033k*μf(cp)f ,τ=(ρcp)p(ρcp)f ,    Pr=μf(cp)fkf ,    ξ=kr2a2μf ,    Sc=μfρfDB ,    β=T1−T0T0 ,    E=EaωT0 ,    Nb=ρfτDB(C1−C0)μf ,    Nt=ρfτDT(T1−T0)μfTm ,    Pe=b1*WeDm ,    Ω=N0N1−N0 ,    UHS=−Exεefξcμf,     κ=aez2n0εefkBTe,    ϕ¯=ϕξ,      u=∂ψ∂y,     v=−δ∂ψ∂x,

The above-mentioned quantities are non-dimensional parameters and their nomenclature is given as follows: θ is the temperature, σ is the nanoparticle volume fraction, χ represents motile microorganisms, M is the Hartmann number, Re is the Reynolds number, δ is the wave number, Rb is the bioconvection Rayleigh constant, Gr is the thermal Grashof number, Nr is the buoyancy ratio constant, Rn is the radiation parameter, τ is effective heat capacity ratio of nanoparticle material to liquid heat capacity, Pr is the Prandtl number, ξ is the reaction rate constant, Sc is the Schmidt number, β is the temperature ratio parameter, E is the activation energy parameter, Nb is the Brownian motion parameter, Nt is the thermophoresis parameter, Pe is the Peclet number, Ω is the concentration difference constant for microorganisms, $U_{HS}$ is the Helmholtz–Smoluchowski velocity and κ is the electro-osmosis parameter.

Using the non-dimensional quantities and transformations, and the assumptions of the lubrication approach, the governing equations can be converted as follows:

(17) $$\frac{\mu_{hnf}}{\mu_{f}}\frac{\partial^{4}\psi }{\partial y^{4}}-\frac{\sigma_{hnf}}{\sigma_{f}}M^{2}\frac{\partial^{2}\psi }{\partial y^{2}}+Gr\left(\frac{{\left(\rho \beta \right)}_{hnf}}{{\left(\rho \beta \right)}_{f}}\frac{\partial \theta }{\partial y}-Nr\frac{\partial \sigma }{\partial y}-Rb\frac{\partial \chi }{\partial y}\right)+\kappa^{3}U_{HS}\left(\left(\frac{\cosh\left(\kappa \left(y+h\right)\right)}{\sinh\left(2h\kappa \right)}\right)\right)=0\;,$$

(18) $$\left(\frac{k_{hnf}}{k_{f}}+Rn\mathrm{Pr}\right)\frac{\partial^{2}\theta }{\partial y^{2}}+Nb\mathrm{Pr}\frac{\partial \theta }{\partial y}\frac{\partial \sigma }{\partial y}+Nt\mathrm{Pr}{\left(\frac{\partial \theta }{\partial y}\right)}^{2}=0\;,$$

(19) $$\frac{\partial^{2}\sigma }{\partial y^{2}}+\frac{Nt}{Nb}\frac{\partial^{2}\theta }{\partial y^{2}}-Sc\xi \sigma {\left(1+\beta \theta \right)}^{n}\exp\left(-\frac{E}{1+\beta \theta }\right)=0\;,$$

(20) $$\frac{\partial^{2}\chi }{\partial y^{2}}-Pe\left(\chi +\Omega \right)\frac{\partial^{2}\sigma }{\partial y^{2}}-Pe\frac{\partial \chi }{\partial y}\frac{\partial \sigma }{\partial y}=0\;,$$

with the following corresponding dimensionless boundary conditions:

(21) $$\psi =\frac{F}{2}\;,\;\;\;\;\frac{\partial \psi }{\partial y}=-1,\;\;\;\;\theta =0\;,\;\;\;\;\;\sigma =0\;,\;\;\;\;\;\chi =0,\;\;\;\;\;\phi =1\;\;\;\;\;\mathrm{at}\;\;\;\;\;y=h\left(=1+\varepsilon \sin\left(x\right)\right),$$

(22) $$\psi =\frac{F}{2}\;,\;\;\;\;\frac{\partial \psi }{\partial y}=-1,\;\;\;\;\;\theta =1\;,\;\;\;\;\;\sigma =1\;,\;\;\;\;\;\chi =1,\;\;\;\;\;\phi =0\;\;\;\;\;\mathrm{at}\;\;\;\;\;y=-h\left(=-(1+\varepsilon \sin\left(x\right))\right)\;,$$

where Q (=F+1) is the time mean flow rate in the fixed frame and $F=\int_{-h}^{h}\left(\partial \psi /\partial y\right)dy$ is the time mean flow rate in the wave frame.

The non-dimensional shear stress, Nusselt number and Sherwood number at the right wall can be represented as follows:

(23) $$\tau_{s}=\frac{\mu_{hnf}}{\mu_{f}}{\left(\frac{\partial u}{\partial y}\right)}_{y=h}\;,$$

(24) $$Nu=\frac{k_{hnf}}{k_{f}}{\left(\frac{\partial h}{\partial x}\frac{\partial \theta }{\partial y}\right)}_{y=h}\;,$$

(25) $$Sh={\left(\frac{\partial h}{\partial x}\frac{\partial \sigma }{\partial y}\right)}_{y=h}\;.$$

## 3. Numerical Procedure

Equations (17–20) with their corresponding boundary conditions (Equations (21,22)) are highly nonlinear and not amenable to find the exact solutions. For this reason, the system of equations was solved with the NDSolve command in Mathematica based on the shooting method. NDSolve is a widely used mathematical solver for the solution of ordinary differential equations and some partial differential equations. This technique can also handle some algebraic differential equations, which are a mix of algebraic and differential equations. NDSolve can solve both initial and boundary value problems iteratively. Nowadays, this technique is widely used by researchers [48,49].

## 4. Results and Discussion

This section deals with the numerical results of velocity, nanoparticle volume fraction, temperature, motile microorganisms, shear stress, Nusselt number and Sherwood number with sundry parameters of the Hartmann number (M), Peclet number (Pe), activation energy parameter (E), thermophoresis parameter (Nt), Schmidt number (Sc), electro-osmosis parameter (κ), Grashof number (Gr), radiation parameter (Rn) and Brownian motion parameter (Nb) in graphical and tabular forms. In Table 1, the thermophysical properties of the base fluid and nanoparticles are provided. The analysis was performed considering the following quantities: $M=2,\;Gr=2,\;Nr=1,\;Rb=2,\;\kappa =2,\;U_{HS}=1,\;Rn=2,\;Pr=6.2,\;Nb=1,\;Nt=2,\;Sc=1,\;\xi =1,\;\beta =1,\;n=0.5,\;E=1,\;Pe=2,\;\chi =2,\;\Omega =0.5,\;\varepsilon =0.2,\;Q=2,\;x=0.1,\;\varphi_{1}=0.0005,\;\varphi_{2}=0.0005.$

**Figure 2:**
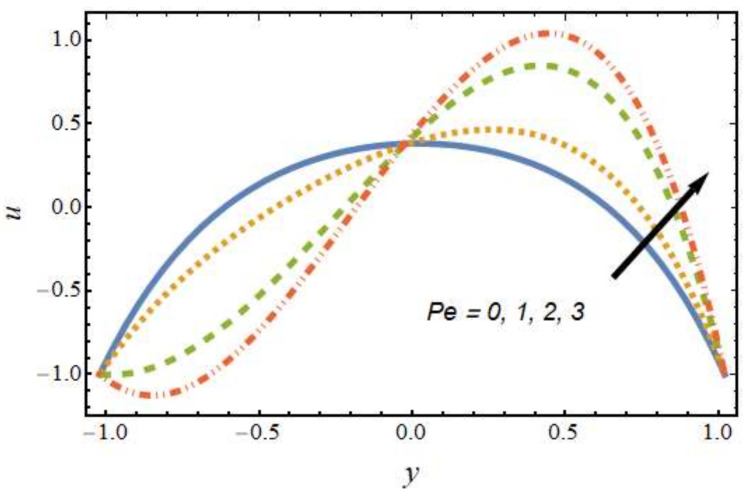
Velocity profile for various values of the Peclet number.

**Figure 3:**
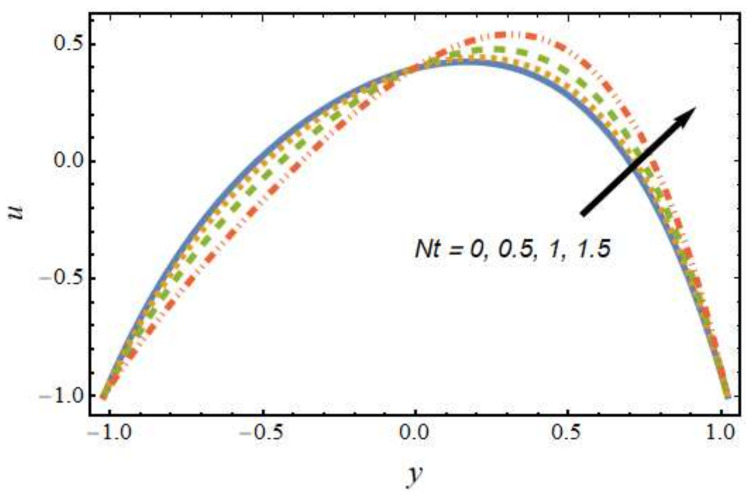
Velocity profile for various values of the thermophoresis parameter.

**Figure 4:**
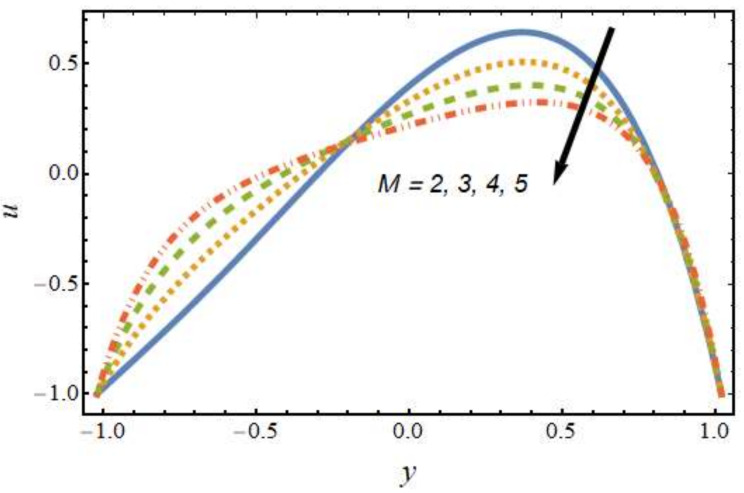
Velocity profile for various values of the Hartmann number.

**Figure 5:**
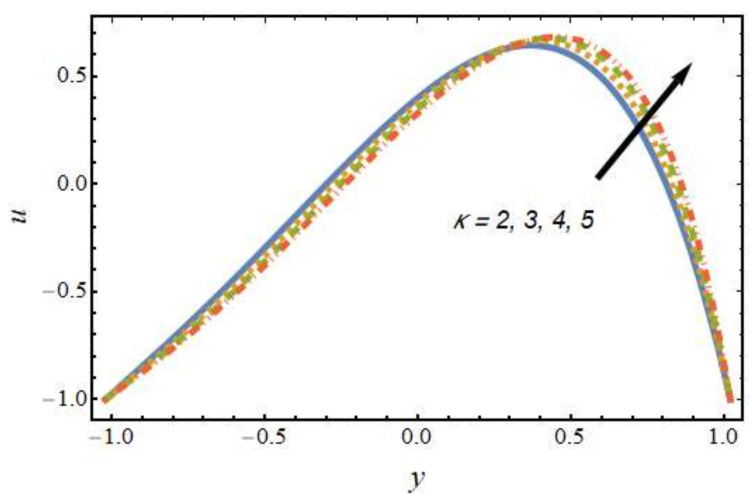
Velocity profile for various values of the electro-osmosis parameter.

**Figure 6:**
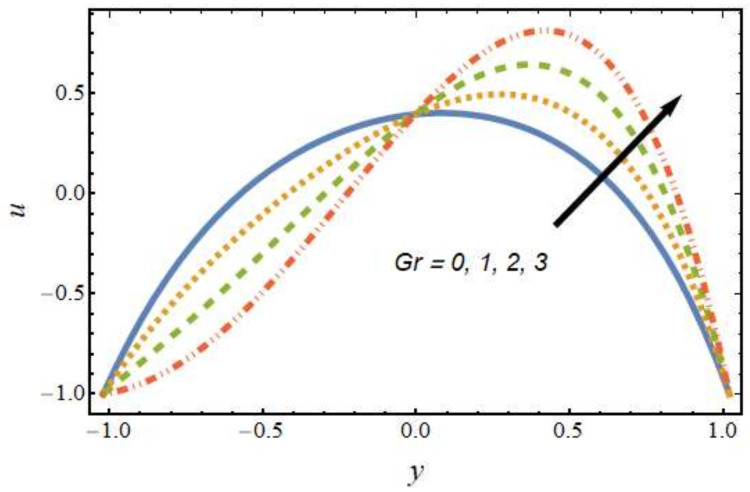
Velocity profile for various values of the Grashof number.

**Figure 7:**
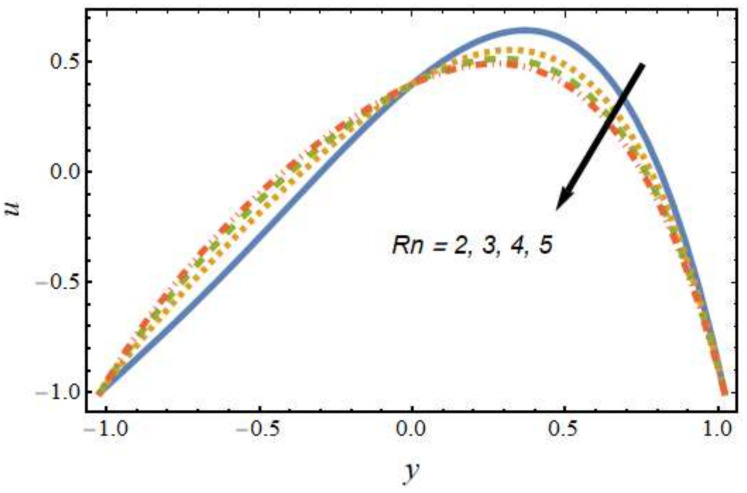
Velocity profile for various values of the radiation parameter.

Figure 2 shows the differences in velocity for various values of the Peclet number. It is evident that the velocity of the hybrid nano-liquid was enhanced with rising values of the Peclet number near the right wall, and the trend was reversed near the left wall. The effects of the thermophoresis parameter on velocity are seen in Figure 3; it can be noted that near the left wall, the hybrid nano-liquid velocity decreased, while it increased near the right wall of the peristaltic channel. The effects of variations in the Hartmann number on velocity are presented in Figure 4; it is clear that the velocity of fluid was decreased in center of the channel and enhanced near the walls of the channel. This is due to the fact that an increasing rate of magnetic field creates a Lorentz force, which acts as a resistive drag force against the motion of the hybrid nanofluid. Figure 5 shows the effects of the electro-osmosis parameter on the velocity of the hybrid nanofluid. It is clear that near the right wall, the velocity of the hybrid nanofluid was enhanced, while it was reduced near the left wall. This is due to the fact that the electrical double layer (EDL) is inversely proportional to the electro-osmotic parameter, which causes hike in the hybrid nanofluid’s velocity near the right wall of the peristaltic channel. Figure 6 displays the velocity variations with respect to the Grashof number; it was observed that the velocity was an increasing function of the Grashof number near the right wall and a decreasing function near the left wall. Physically, an enhancement in the Grashof number increases the thermal energy of the fluid molecules and loosens up intermolecular forces within the fluid particles, which means the fluid is less viscous due to an increase in temperature. Figure 7 shows the variations in velocity with an increase in the radiation parameter; it should be noted that the velocity was enhanced near the left wall and the trend was reversed near the right wall of the peristaltic channel.

**Figure 8:**
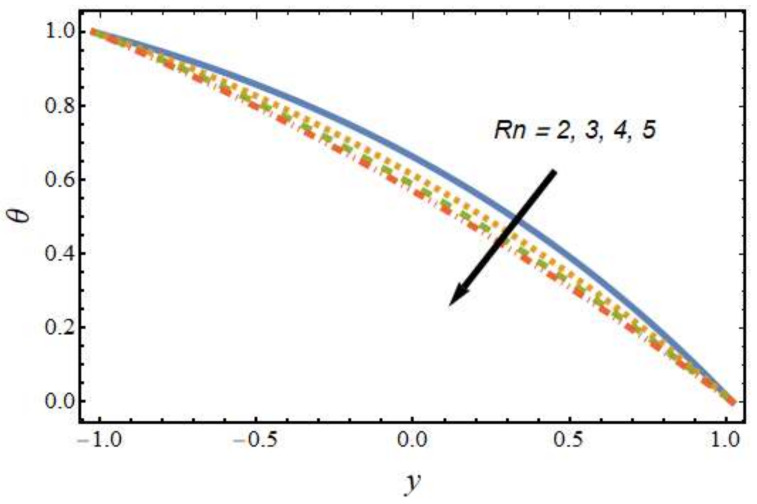
Nanoparticle temperature profile for various values of the radiation parameter.

**Figure 9:**
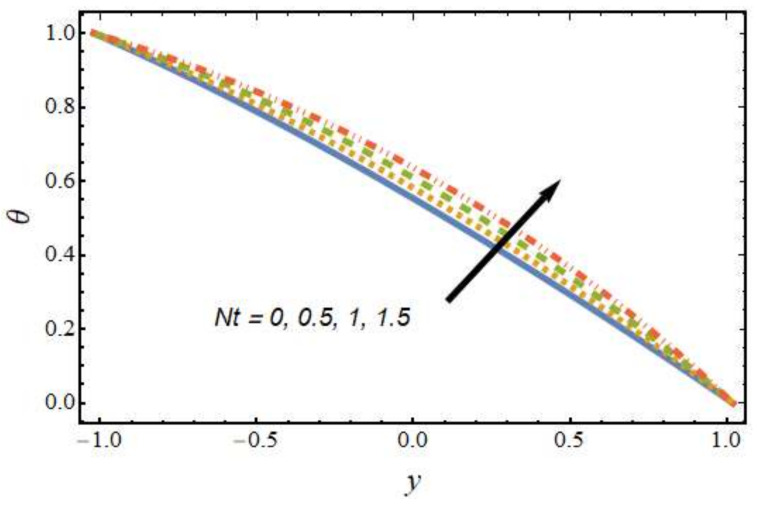
Nanoparticle temperature profile for various values of the thermophoresis parameter.

**Figure 10:**
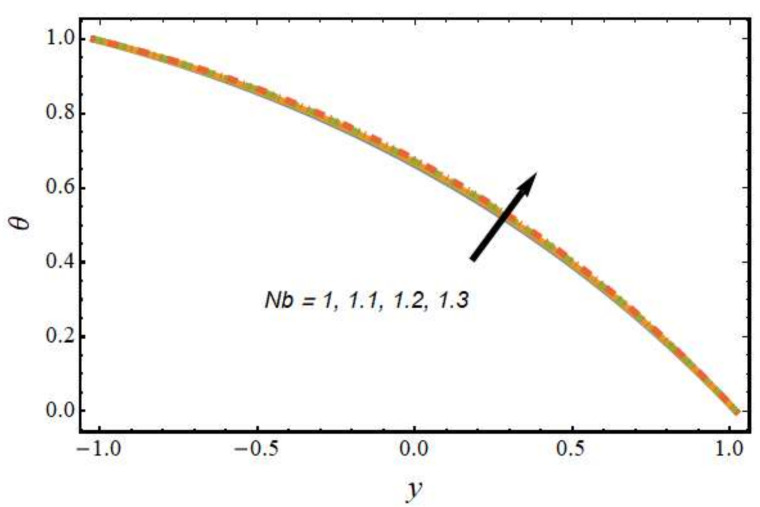
Nanoparticle temperature profile for various values of the Brownian motion parameter.

**Figure 11:**
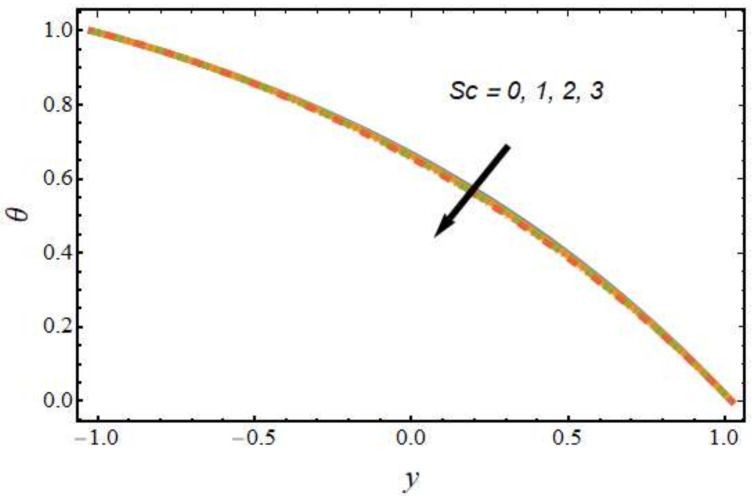
Nanoparticle temperature profile for various values of the Schmidt number.

**Figure 12:**
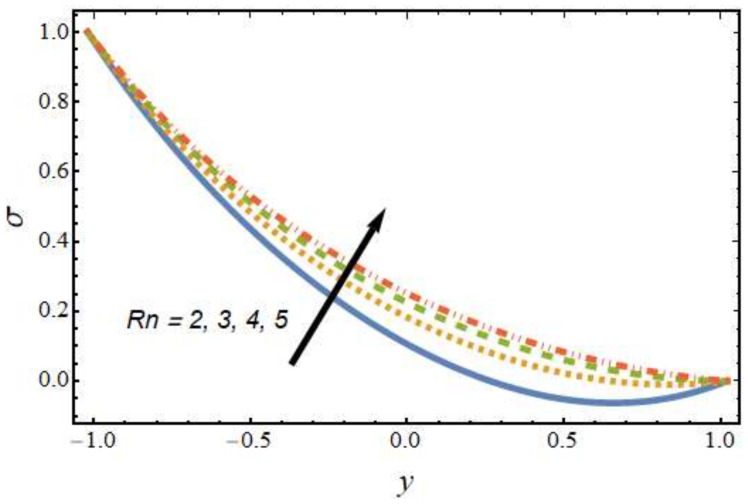
Nanoparticle concentration profile for various values of the radiation parameter.

**Figure 13:**
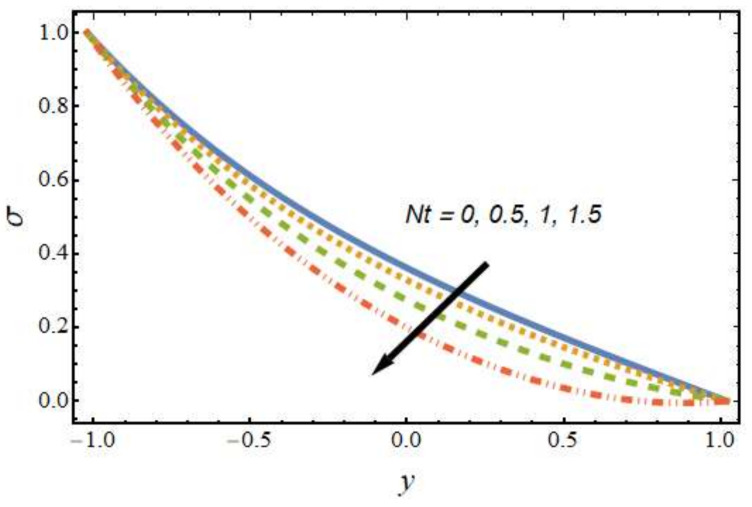
Nanoparticle concentration profile for various values of the thermophoresis parameter.

**Figure 14:**
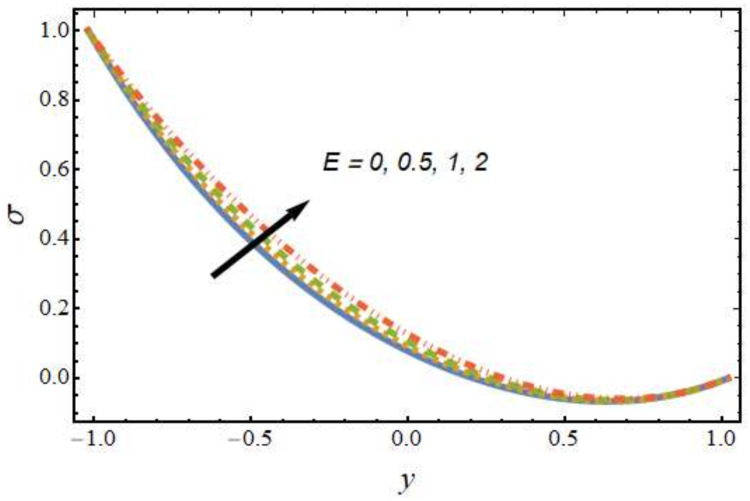
Nanoparticle concentration profile for various values of the activation energy parameter.

**Figure 15:**
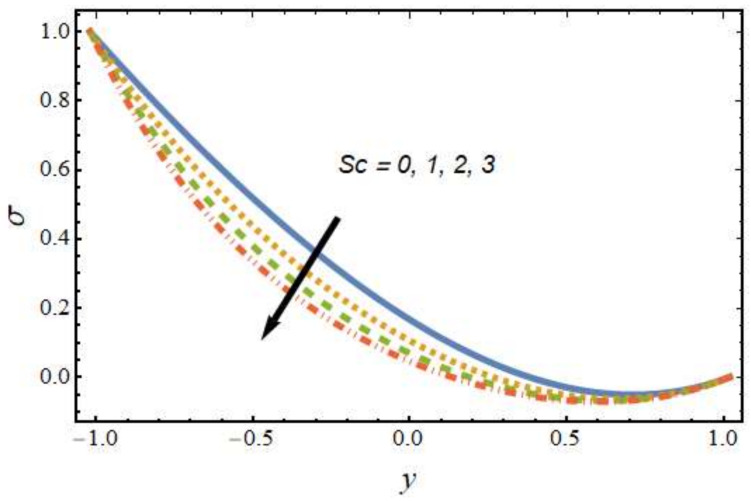
Nanoparticle concentration profile for various values of the Schmidt number.

**Figure 16:**
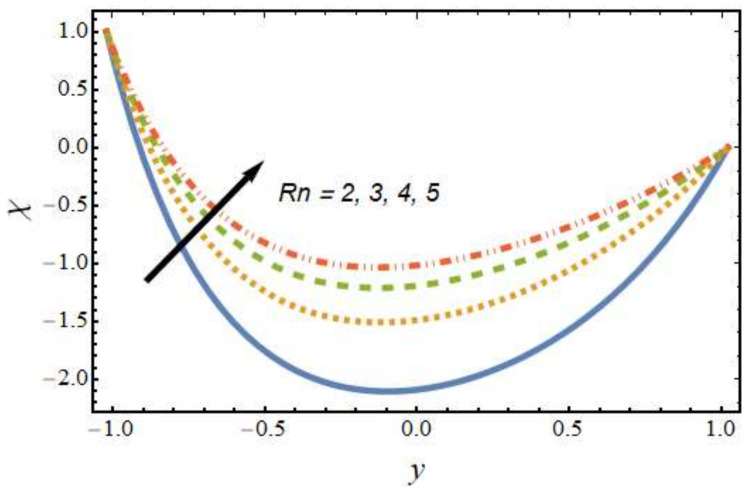
Motile microorganism profile for various values of the radiation parameter.

**Figure 17:**
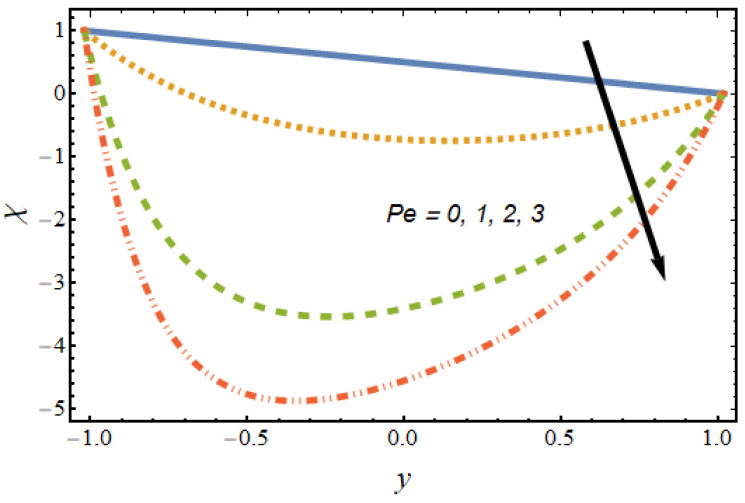
Motile microorganism profile for various values of the Peclet number.

**Figure 18:**
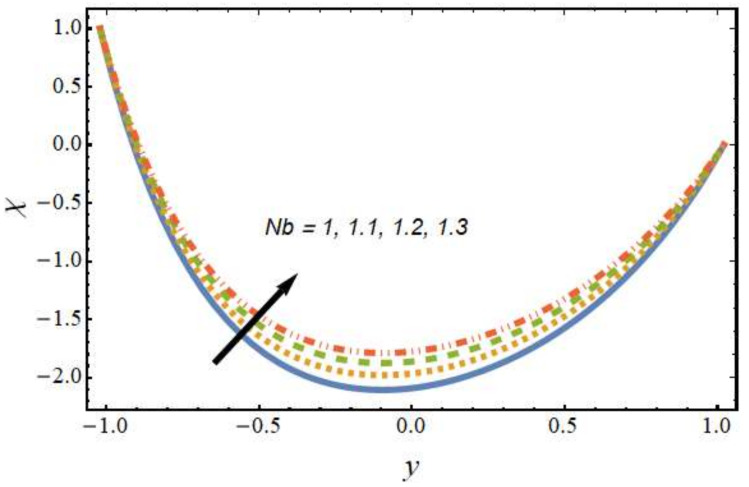
Motile microorganism profile for various values of the Brownian motion parameter.

**Figure 19:**
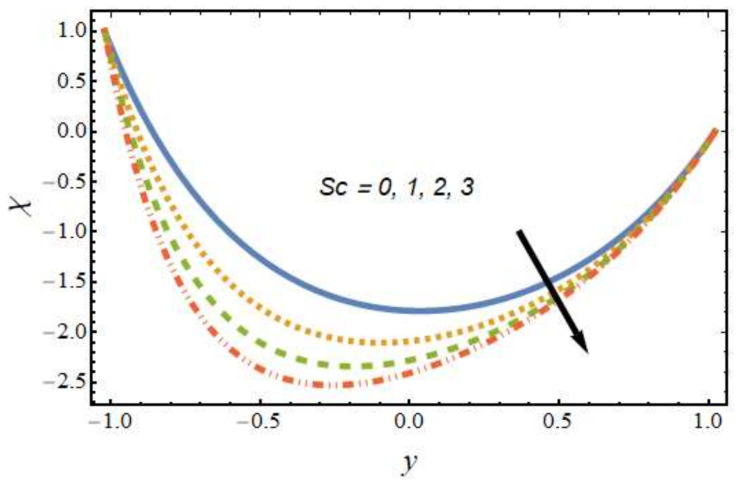
Motile microorganism profile for various values of the Schmidt number.

Figure 8, Figure 9, Figure 10 and Figure 11 show the nanoparticle temperature for the various parameters involved, including the radiation parameter, Schmidt number, thermophoresis parameter and Brownian motion parameter. The nanoparticle temperature decreased with rising values of the radiation parameter (Figure 8). Physically, the higher value of radiation effects leads to an increase in the dominance of conduction over radiation. Hence, a decline in thermal boundary layer thickness and buoyancy force is observed, and this results in a decrease in nanoparticle temperature. It can be observed from Figure 9 that the nanoparticle temperature was enhanced with an increase in the thermophoresis parameter. The thermophoresis phenomenon is based on the migration of nanoparticles from a hot zone to a cold zone due to the temperature difference. Figure 10 shows that the nanoparticle temperature is an increasing function of Brownian motion parameter. This is due to the rotations and vibrations of molecules with the kinetic energy of molecular Brownian motion. The nanoparticle temperature decreased with an increase in the Schmidt number (see Figure 11). The consequences of the radiation parameter, thermophoresis parameter, Schmidt number and activation energy on the nanoparticle concentration in the hybrid nanofluid are illustrated in Figure 12, Figure 13, Figure 14 and Figure 15. The impression of the radiation parameter on the nanoparticle concentration field of the hybrid nanofluid is exhibited in Figure 12. It should be noted that rising radiation effects increased the nanoparticle concentration field. It can be observed in Figure 13 that the nanoparticle concentration profile declined with an increase in the thermophoresis parameter for the hybrid nanofluid. The influence of activation energy on the hybrid nanofluid nanoparticle concentration profile is represented in Figure 14. Increasing activation energy induced an increase in the nanoparticle volume fraction. The Arrhenius function decays by increasing the value of the activation energy, which results in the promotion of the generative chemical reaction causing an augmentation in the nanoparticle concentration profile. It can be seen from Figure 15 that the nanoparticle concentration profile declined with rising values of the Schmidt number for the current hybrid nanofluid. Physically, as the Schmidt number rises, the kinematic viscosity also increases, which in turn reduces molecular diffusion; hence, the reduction in mass is observed. Figure 16, Figure 17, Figure 18 and Figure 19 represent the effects of the radiation parameter, Peclet number, Brownian motion parameter and Schmidt number on the dimensionless motile microorganism profile of the hybrid nanofluid. Figure 16 shows that the value of the radiation parameter increased the motile microorganism distribution. Figure 17 displays the deviation between motile microorganisms and the Peclet number. It should be noted that the Peclet number reduced the profile of motile microorganisms. Figure 18 represents the influence of the Brownian motion parameter on the motile microorganism profile of the hybrid nanofluid. It should be noted that the motile microorganism profile increased with the Brownian motion parameter. Figure 19 represents a decline motile microorganisms with rise in the Schmidt number. Table 2 represents the variations in the shear stress, Nusselt number and Sherwood numbers with the various involved fluid parameters. Common observations from the table indicate that the shear stress was enhanced with the activation energy, radiation parameter and Brownian motion parameter; in the case of the fitted rate, thermophoresis parameter and Schmidt number, it declined. The Nusselt number was enhanced with the radiation parameter, fitted number and Schmidt number, and this trend was opposed for the activation energy, Brownian motion parameter and thermophoresis parameter. The fitted number, thermophoresis parameter and Schmidt number increased the Sherwood number, which decreased with an increase in the activation energy, radiation parameter and Brownian motion parameter. Table 3 shows a comparison of the present study with the existing literature [52]; for comparison, we considered the fixed values of Gr, Nr, Nb, Nt, ε and x in both cases and the other involved parameters were kept at zero in the present analysis. It can be concluded from the table that the present results are in good agreement with the existing literature.

## 5. Conclusions

The current article dealt with the propulsion of a gold–silver–water hybrid nanofluid flow through a peristaltic microchannel under the effects of activation energy, radiation, Brownian motion, a magnetic field and an electric field. Initially, the nonlinear system of equations was simplified under the LWL and LRN approximations. The resulting highly nonlinear system was solved with the computational software Mathematica. Finally, the numerical results were provided for the Sherwood number, Nusselt number, shear stress, motile microorganisms, nanoparticle volume fraction, nanoparticle temperature and velocity with sundry parameters in pictorial and tabular forms. It should be noted from Table 3 that the limiting cases of the current analysis are in good agreement with the existing literature [52]. The main findings of the current investigation are as follows:

1. The velocity enhances with the Peclet number, thermophoresis parameter, electro-osmosis parameter and Grashof number near the right wall of the peristaltic channel.
2. Stronger magnetic strengths reduce the velocity of the hybrid nanofluid.
3. Stronger thermophoresis and Brownian motion effects lead to an increment in nanoparticle temperature.
4. The radiation and Schmidt effects decrease the temperature profile.
5. Nanoparticle concentration is enhanced with the radiation parameter and activation energy.
6. The radiation and Brownian effects uplift the motile microorganism profiles.
7. Shear stress increases with the enhancement of activation energy and radiation effects, and the trend is reversed for the thermophoresis and Schmidt parameters.
8. The radiation and Brownian motion effects increase the motile microorganism profiles.
9. The Sherwood number and Nusselt number are decreasing functions of the activation energy and Brownian motion effects, and the trend is reversed for the Schmidt number and fitted parameter.

The findings of the present mathematical analysis will be a benchmark for simulating a more generalized model in three-dimensions for hybrid nanofluid flow with various geometries of nanoparticles in blood vessels, such as arteries and capillaries, for the better visualization and real applications of drug delivery in the circulatory system.

## Figures and Tables

**Table 1:** Thermophysical properties of nanoparticles and base fluid [50,51].

Properties	Gold	Silver	Water
$k\left(W/\mathrm{mK}\right)$	318	429	0.613
$c_{p}\left(J/\mathrm{kgK}\right)$	129	235	4179
$\rho \left(\mathrm{kg}/m^{3}\right)$	19,300	10,500	997.1
σ(S/m)	$4.1\times 10^{6}$	$3.6\times 10^{7}$	0.05
$\beta \left(1/k\right)\times 10^{-5}$	1.4	1.89	21

**Table 2:** Variations in Nusselt number, shear stress, and Sherwood number with various fluid parameters.

E	Rn	n	Nb	Nt	Sc	$\tau_{s}$	Nu	Sh
1	2	0.5	1	2	1	−6.3641	−0.1816	0.0744
1.5						−6.3148	−0.1823	0.0738
2						−6.2727	−0.1829	0.0732
2.2						−6.2579	−0.1831	0.0730
	3.0					−5.7522	−0.1522	0.0226
	3.1					−5.7135	−0.1504	0.0194
	3.2					−5.6774	−0.1487	0.0164
	3.3					−5.6435	−0.1471	0.0136
		0.1				−6.3182	−0.1823	0.0736
		0.2				−6.3290	−0.1821	0.0738
		0.3				−6.3402	−0.1820	0.0740
		0.4				−6.3519	−0.1818	0.0742
			0.5			−7.8738	−0.1685	0.1689
			0.6			−7.3268	−0.1711	0.1373
			1.1			−6.2456	−0.1843	0.0659
			1.2			−6.1492	−0.1870	0.0589
				0		−4.6605	−0.1200	−0.0671
				0.3		−4.7555	−0.1280	−0.0598
				0.6		−4.8972	−0.1364	−0.0482
				1		−5.1673	−0.1483	−0.0254
					0	−6.1092	−0.1852	0.0699
					1	−6.3641	−0.1816	0.0744
					1.2	−6.4020	−0.1811	0.0748
					2	−6.5249	−0.1794	0.0757

**Table 3:** Comparison of velocity profile with the existing literature [52].

y	Present Study	Existing Literature [52]
−1	−1.0000	−1.0000
−0.8	−0.4599	−0.4600
−0.6	−0.0399	−0.0400
−0.4	0.2600	0.2600
−0.2	0.4400	0.4400
0	0.5000	0.5000
0.2	0.4400	0.4400
0.4	0.2600	0.2600
0.6	−0.0399	−0.0400
0.8	−0.4599	−0.4600
1	−1.0000	−1.0000

## Data Availability

The data that support the findings of this study are available from the corresponding author upon reasonable request.
